# BN/Ag hybrid nanomaterials with petal-like surfaces as catalysts and antibacterial agents

**DOI:** 10.3762/bjnano.9.27

**Published:** 2018-01-23

**Authors:** Konstantin L Firestein, Denis V Leybo, Alexander E Steinman, Andrey M Kovalskii, Andrei T Matveev, Anton M Manakhov, Irina V Sukhorukova, Pavel V Slukin, Nadezda K Fursova, Sergey G Ignatov, Dmitri V Golberg, Dmitry V Shtansky

**Affiliations:** 1 National University of Science and Technology “MISIS”, Leninsky prospect 4, Moscow, 119049, Russian Federation; 2 School of Chemistry, Physics and Mechanical Engineering, Science and Engineering Faculty, Queensland University of Technology (QUT), 2nd George st., Brisbane, QLD 4000, Australia; 3 State Research Center for Applied Microbiology and Biotechnology, Obolensk, Moscow Region 142279, Russian Federation; 4 Moscow State University, Department of Geocryology, Moscow 119992, Russian Federation; 5 World Premier International Center for Materials Nanoarchitectonics (WPI-MANA), National Institute for Materials Science (NIMS), Tsukuba, Namiki 1, Ibaraki 3050044, Japan

**Keywords:** antibacterial agents, BN/Ag hybrid nanomaterials, catalysts, chemical vapour deposition, nanomaterials

## Abstract

BN/Ag hybrid nanomaterials (HNMs) and their possible applications as novel active catalysts and antibacterial agents are investigated. BN/Ag nanoparticle (NP) hybrids were fabricated using two methods: (i) chemical vapour deposition (CVD) of BN NPs in the presence of Ag vapours, and (ii) ultraviolet (UV) decomposition of AgNO_3_ in a suspension of BN NPs. The hybrid microstructures were studied by high-resolution transmission electron microscopy (HRTEM), high-angular dark field scanning TEM imaging paired with energy dispersion X-ray (EDX) mapping, X-ray photoelectron spectroscopy (XPS), and infrared spectroscopy (FTIR). They were also characterized in terms of thermal stability, Ag^+^ ion release, catalytic and antibacterial activities. The materials synthesized via UV decomposition of AgNO_3_ demonstrated a much better catalytic activity in comparison to those prepared using the CVD method. The best catalytic characteristics (100% methanol conversion at 350 °C) were achieved using the UV BN/Ag HNMs without preliminary annealing at 600 °C in an oxidizing atmosphere. Both types of the BN/Ag HNMs possess a profound antibacterial effect against *Escherichia coli* K-261 bacteria.

## Introduction

New hybrid nanomaterials are the key components of the next generation advanced catalysts and biomaterials. Novel and unique properties can be obtained while employing synergetic effects of different nanocomponents. In recent years, BN nanostructures have been in the focus due to advantageous combination of properties, such as high tensile strength and elastic modulus, superb chemical stability, biocompatibility, high thermal conductivity and perfect electrical insulation. This explains their rich functionality in reinforcement of ultralight metals and ceramics, improvement of thermal conductivity and mechanical strength of diverse polymers, production of transparent superhydrophobic films, and quantum electronic and photonic devices [1–4]. BN nanomaterials have also been utilized toward the development of cutting-edge hybrid nanostructures. For example, BN/noble metal (Pt, Au, Ag) hybrid nanomaterials are envisaged to be the promising components of highly active catalysts, drug delivery systems, molecular probe sensors, surface enhanced Raman spectroscopy techniques, and antibacterial agents [5–10].

Herein, we have focused on the fabrication of BN/Ag hybrid nanomaterials (HNMs) and their emerging applications as novel highly active catalysts and antibacterial agents. It has previously been shown in several theoretical [11] and experimental studies [5,12–14] that BN/Ag hybrid nanomaterials may possess high activity and stability as catalysts for oxidation of organic compounds. Moreover, N-impurities and other defects in thin layers of raw *h*-BN can act as active centers for oxygen reduction reactions [15]. Generally, the catalytic activity of BN/Ag hybrid nanomaterials strongly depends on the structure and imperfection of *h-*BN supports. Thus, we have utilized BN nanoparticles (BN NPs) with a “pompon”-like structure (i.e., petalled BN NPs) as a base material for creating BN/Ag hybrid nanomaterials. Due to rough surface and a large number of 2D BN nanosheet petals these BN NPs are thought to demonstrate advanced characteristics as catalyst supports. Also, colloidal silver is effective against different types of microorganisms, therefore BN/Ag HNMs are of great interest as antibacterial agents. For example, it has been reported by Gao et al. [10] that the bactericidal properties of *h*-BN nanosheets can be significantly improved after the growth of Ag nanoparticles (NPs) on their surfaces.

In our previous work [16] we have produced BN/Ag HNMs by Ag ion implantation into the BN NPs with petal-like surfaces. However, this technique does not allow for the production of these nanostructures in an amount sufficient for detailed catalytic and biological tests. Therefore, in the present work, we have synthesized BN-based HNMs using two high-throughput methods: (i) chemical vapour deposition (CVD) of BN NPs in the presence of Ag vapors; and (ii) ultraviolet (UV) decomposition of AgNO_3_ in a suspension of BN NPs. Both methods resulted in partial oxidation of Ag during processing. Then, the obtained materials have been characterized in terms of their microstructures, thermal stability, Ag^+^ ion release, and catalytic and antibacterial activities.

## Results and Discussion

### Structure of as-synthesized BN/Ag(O) hybrid nanomaterials

**Figure 1:**
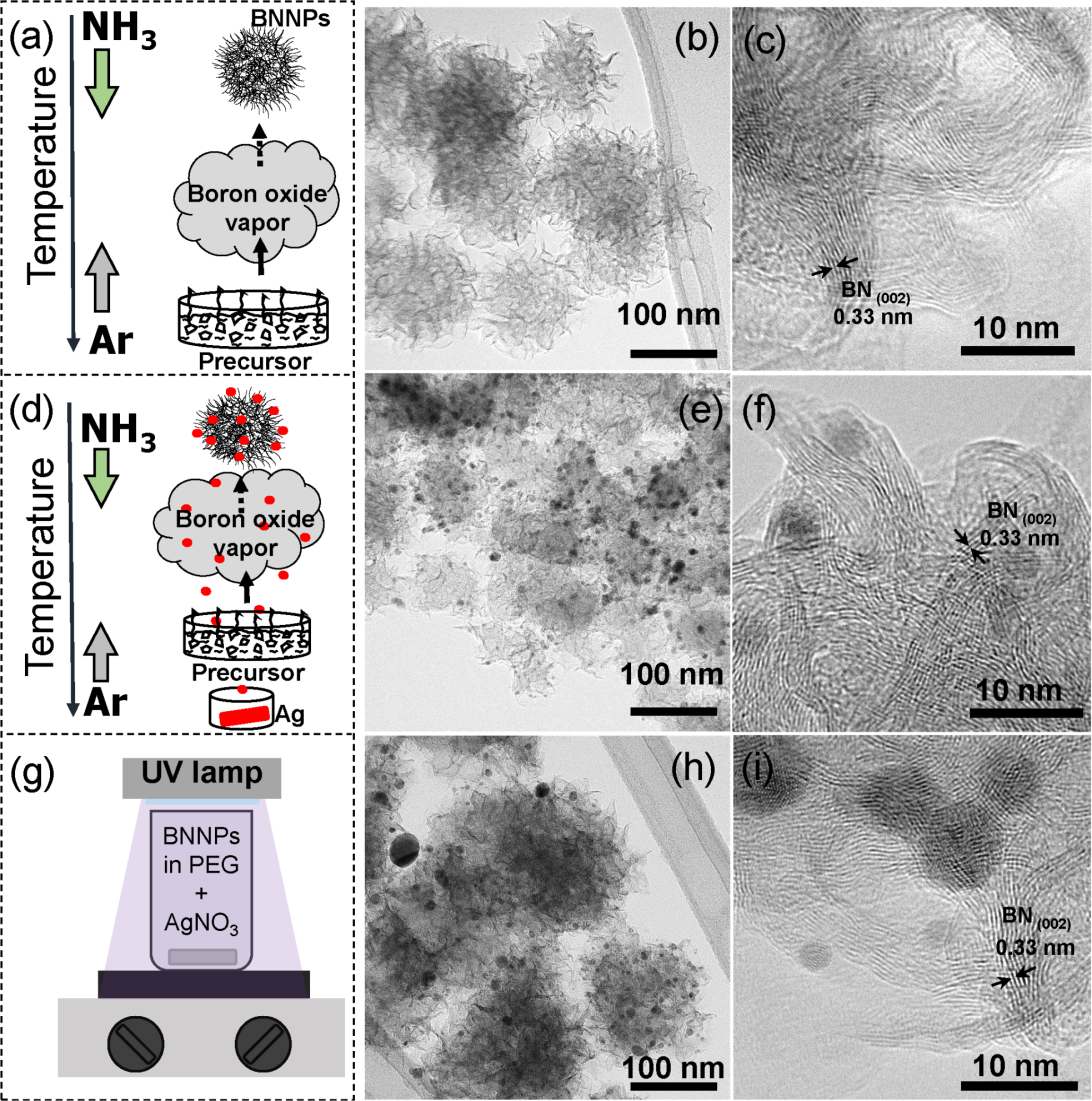
Formation schemes of BN NPs (a) and BN/Ag hybrid nanomaterials via CVD (d) and UV decomposition of AgNO_3_ (g) together with the corresponding low-magnification TEM images (b, e, h) and the corresponding high-resolution TEM images (c, f, i).

The preparation processes of pure BN NPs and both types of the BN/Ag HNMs together with their TEM images are illustrated in Figure 1. Raw BN NPs with an average size of 100–200 nm can be described as “pompon”-like or petalled structures, i.e., the solid particles consisting of numerous BN nanosheets, 30–50 nm in length and 10–25 nm in width, with a characteristic interplanar spacing of 0.33 nm (Figure 1c).

The formation of Ag NPs on the BN NP surfaces during the syntheses did not lead to a significant modification of the initial BN NP structures (Figure 1e and 1h). For both sample types, Ag NPs, 5–15 nm in size, were observed to be uniformly distributed over the BN NP surfaces. The morphology of the BN/Ag HNMs and the average size of Ag NPs, prepared by two different methods, were similar, although for the UV BN/Ag samples, some larger Ag NPs, up to 35 nm, were also detected (Figure 1h). The HRTEM images of individual BN/Ag HNMs obtained via CVD and UV decomposition methods are illustrated in Figure 1f and 1i. The outer BN NP surface is formed by BN nanosheets consisting of several *h*-BN atomic layers and Ag NPs located between the petals.

**Figure 2:**
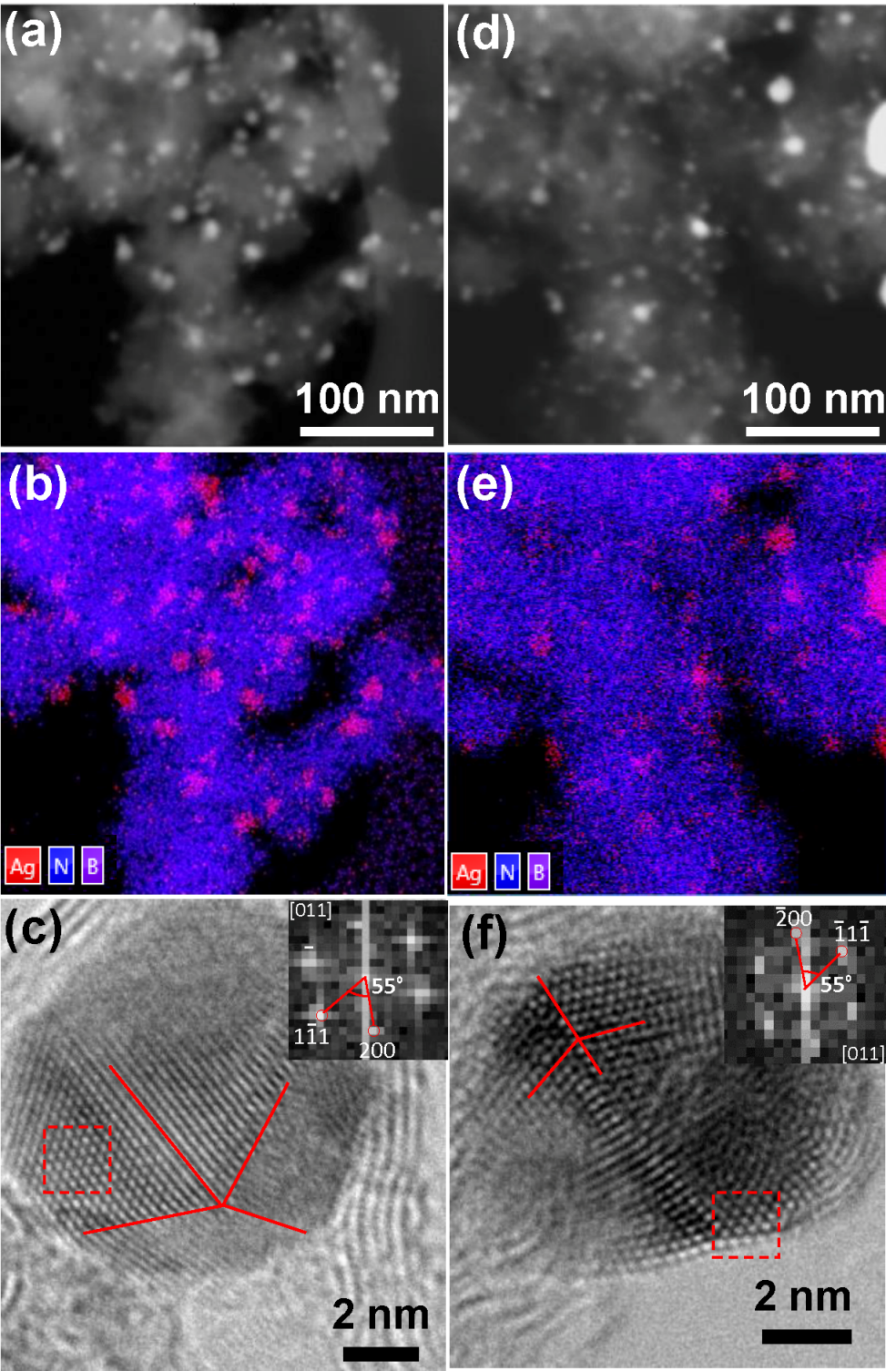
HADF-STEM (a, d) images together with the corresponding spatially-resolved EDX maps (b, e) of BN/Ag HNMs produced via CVD (a, b, c) and UV decomposition of AgNO_3_ (d, e, f). HRTEM images of individual Ag NPs showing their atomic structure (c, f), FFT patterns from the selected areas are shown in the insets (c, f).

HADF-STEM and spatially-resolved EDX mapping (Figure 2) demonstrate that the surface of BN NPs is densely populated with Ag NPs.

Thorough structural characterization of individual Ag NPs revealed their fine structure. The HRTEM images of individual Ag NPs are depicted in Figure 2c and 2f. The particles with a size smaller than 6–9 nm consisted of small crystallites with coherent (twinned) or semi-coherent grain boundaries. The interplanar distances estimated from the fast Fourier transform (FFT) patterns (Figure 2c and 2f, insets) were *d* = 0.231 nm and *d* = 0.205 nm. These distances well correspond with the (200) and (111) plane separations of the face-centered cubic (FCC) silver (*d*_(200)_ = 0.232 nm and *d*_(111)_ = 0.204 nm). In addition, the angles between spots in FFT images are 55° that further confirms FCC structure of NPs.

In the first approximation, the specific surface area of Ag NPs on BN/Ag NH surfaces can be simply estimated from TEM images using a secant method. Our estimates show that the surface area for Ag NPs per g of silver increases from 2.7 × 10^5^ cm^2^/g for UV BN/Ag HNMs to 4.3 × 10^5^ cm^2^/g for CVD BN/Ag HNMs. Elemental composition of BN/Ag HNMs was determined by EDX analysis and gave the following Ag contents: CVD BN/Ag HNMs – 1.5 wt %, UV BN/Ag HNMs – 2.4 wt %.

**Figure 3:**
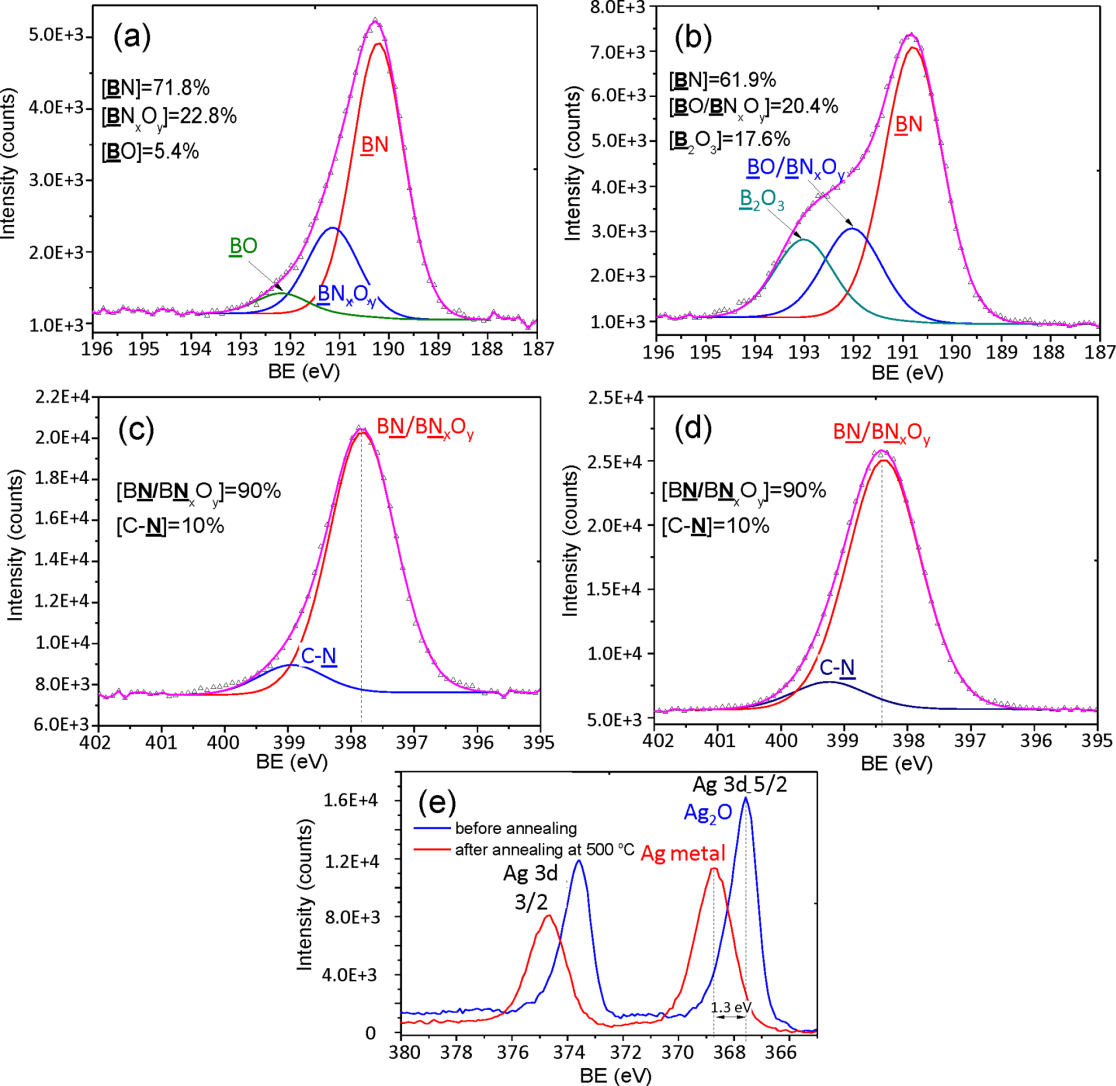
XPS B 1s (a, b), N 1s (c, d), and Ag 3d (e) spectra of BN/Ag hybrid nanomaterials produced via UV decomposition of AgNO_3_ and their curve-fitting (a, c) before and (b, d) after annealing in air at 500 °C for 1 h. (e) Overlay of Ag 3d signal before and after annealing.

To shed a light on surface chemistry of BN/Ag HNMs and to also understand how it changes during heating in the oxidizing atmosphere, we have performed XPS analysis of UV BN/Ag HNMs before and after annealing in air at 500 °C for 1 h. The results of XPS measurements are shown in Table 1. The analysis revealed that BN was the main phase, as evidenced by the **B**N component at the binding energy (BE) of 190.2 eV at a concentration of 71.8 ± 4.0%, as determined after B 1s curve-fitting (Figure 3a). Note also the presence of a small amount of a BN_x_O_y_ phase (BE = 191.2 eV) and a marginal amount of BO (BE = 192.5 eV). The XPS N 1s spectrum shows the peak centred at 397.8 eV, hereby indicating the B–N bonds (Figure 3c). The XPS Ag 3d spectrum revealed that silver was partly in its oxidized state, and the Ag_2_O phase was also apparent within the BN/Ag HNMs (Figure 3e).

**Table 1:** Elemental composition of UV BN/Ag hybrid nanomaterials (C content is subtracted).

Sample	Elemental composition, atom %			
	Ag	B	N	O

before annealing	4.8	40.9	31.5	22.8
after annealing	2.2	44.2	27.6	26.0

### Thermal stability of BN/Ag hybrid nanomaterials

**Figure 4:**
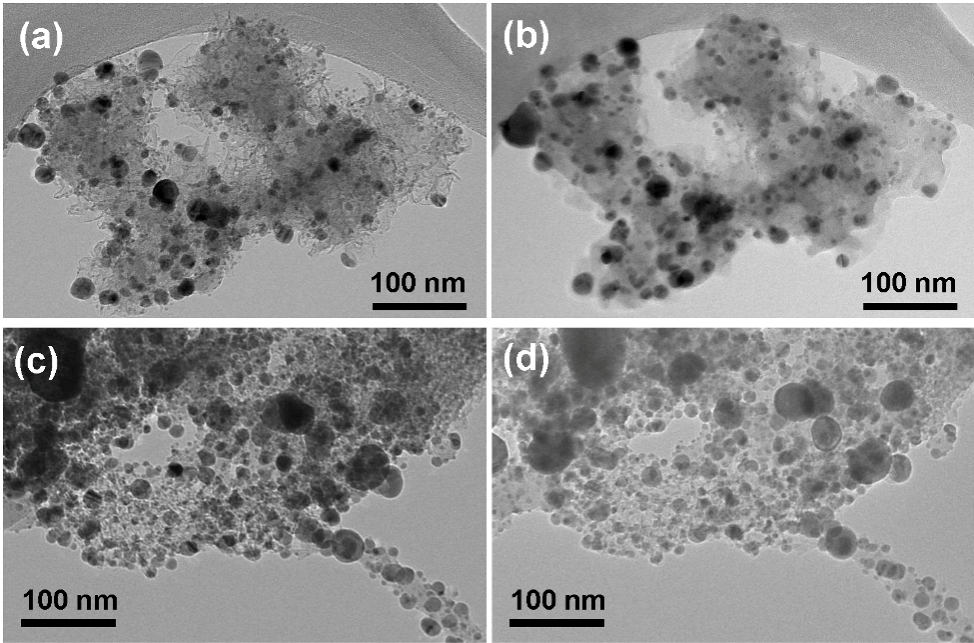
TEM micrographs of BN/Ag HNMs: (a) fabricated via UV decomposition of AgNO_3_, (b) under in situ heating in the TEM column to 700 °C, (c, d) after preliminary annealing in air at 600 °C for 1 h followed by in situ heating in the TEM column at (c) 100 °C and (d) 650 °C.

The majority of NH applications, including catalysts, are closely related to their high-temperature performance. Thus, HNMs with high thermal stability, i.e., capable of maintaining their structure at elevated temperatures, are required. To completely exclude the possible influence of an oxidation process on the phase composition and to directly observe the structural evolutions upon heating, the annealing tests were first conducted directly in the TEM column. Figure 4a and 4b represent TEM micrographs taken from the same area of pristine UV BN/Ag HNMs at room temperature and at 700 °C. We did not observe any structural changes. The NPs retained their size and the distribution pattern over the BN NPs surface, hereby indicating high thermal stability of the HNMs.

In the following experiment we modeled the temperature and environmental conditions during catalytic activity tests (without taking into account a reaction with methanol). For this, the UV BN/Ag HNMs samples were preliminary annealed in air at 600 °C for 1 h and then analyzed inside TEM during additional in situ heating (Figure 4c, d). No any noticeable changes with respect to Ag NPs size were observed.

XPS analyses performed after the sample pre-annealing in air showed that the B, N, and Ag spectra had significantly changed. The Ag 3d spectrum revealed that the Ag 3d_5/2_ and Ag 3d_3/2_ peaks shifted by 1.3 eV to higher BEs, hereby indicating that the Ag_2_O phase decomposed to metallic Ag (Figure 3e). After annealing, the B 1s spectrum exhibited a significant amount of the B_2_O_3_ phase (BE = 193.0 eV). Since the **B**N (BE = 190.8 eV) and **B**O/**B**N_x_O_y_ (BE = 192.0 eV) peaks shifted towards higher BEs, it is reasonable to assume that their neighbors had additionally been oxidized (Figure 3b). The oxidation of BN was also confirmed by N 1s curve-fitting, because the XPS N 1s peak at 397.8 eV shifted to 398.4 eV (Figure 3d).

An important issue that deserves additional comments is the role of Ag in the oxidation of the BN phase during heat treatment, which, as known, can withstand temperatures in excess of 900 °C without chemical degradation [17]. Since Ag_2_O decomposes at temperatures above 280 °C, it is reasonable to assume that Ag NPs (formed during decomposition) possess high activity in oxygen reduction reaction being catalysts at elevated temperatures. The overall chemical reactions within BN/Ag HNMs can be presented in the following simplified forms:

[1]
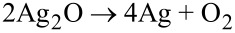


[2]
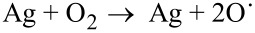


[3]
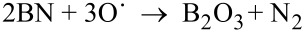


Where reaction 1 represents the decomposition of silver oxide, reaction 2 corresponds to oxygen activation, and reaction 3 shows the BN oxidation.

### Catalytic activity

**Figure 5:**
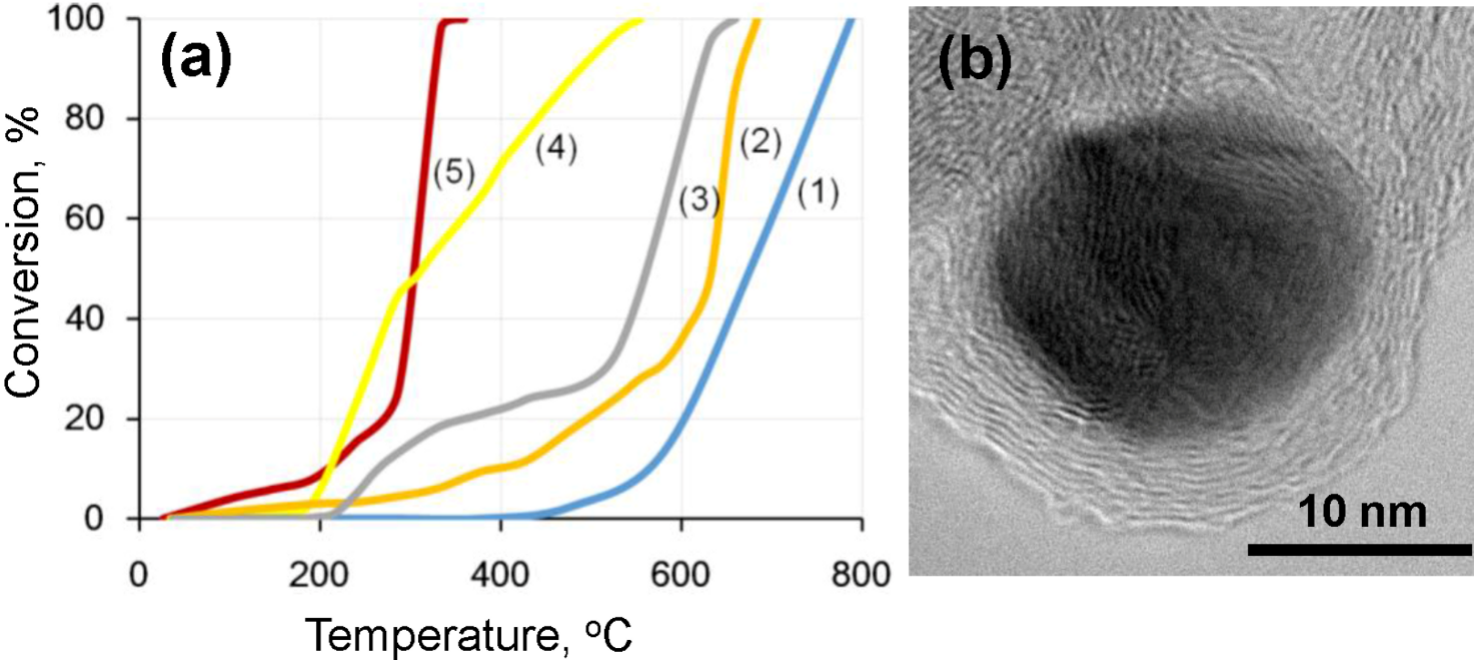
The results of catalytic oxidation of methanol (a) and HRTEM image of Ag NP inside a BN/Ag NH produced via CVD (b). 1 – quartz pellets, 2 – raw BN NPs, 3 – BN/Ag HNMs produced by CVD method, 4 and 5 – BN/Ag HNMs obtained via UV decomposition of AgNO_3_ with (4) and (5) without preliminary activation stage.

The results of catalytic oxidation of methanol for both types of BN/Ag hybrid nanomaterials are presented in Figure 5a. Quartz pellets (curve 1) and raw BN NPs (curve 2) were used as the reference samples. The UV BN/Ag HNMs demonstrated much better catalytic activity (curve 4) in comparison to hybrid nanomaterials prepared using the CVD method (curve 3). In the former case, the methanol conversion reached 50% at 300 °C, whereas in the latter case such a percentage of conversion was only achieved at 550 °С. The conversion became 100% at 530 °C and 650 °C, respectively. Since structural studies revealed a relatively high amount of oxygen, which was additionally increased during preliminary heating, it was suggested that the activation stage is not needed or even harmful due to the formation of a liquid B_2_O_3_ phase at the annealing temperatures. Indeed, the best catalytic characteristics were achieved on the UV BN/Ag HNMs without the activation stage. In this case, the conversion reached 100% already at 350 °C (curve 5).

The observed difference in conversion rate between CVD and UV BN/Ag HNMs can be explained as follows. During the CVD process, the formation of Ag NPs occurred simultaneously with the growth of BN petals. Thus, the major part of Ag NPs was covered with the layers of *h*-BN (Figure 5b). In contrast, during UV decomposition of AgNO_3_, Ag NPs were nucleated on the surface of BN NPs and, therefore, remained free from the *h*-BN shells. Note that CVD BN/Ag HNMs, in which the Ag NPs were predominantly surrounded by a thin layer of *h*-BN, demonstrated the catalytic activity higher than that of the raw BN NPs. This result indicates that BN-shielded Ag NPs still possess a significant catalytic activity. Although their catalytic activity is lower than that of uncovered Ag NPs, one may expect much higher stability of their catalytic properties. Interestingly, the catalytic activity of pure BN NPs was higher compared with inert quartz spheres. This result supports the conclusion of Lyalin et al. [15], who suggested that the impurities and other defects in a *h*-BN structure enhance the catalytic activity of *h*-BN compared with commonly used catalyst supports (i.e., SiO_2_, Al_2_O_3_, etc.).

An important question that needs additional analysis is the role of catalyst support morphology. In contrast to the previous works, that were focused on the catalytic activity of 2D BN nanosheets decorated with Ag NPs during *p*-nitrophenol reduction to *p*-aminophenol, i.e., for liquid phase reaction [12–14], we utilized the 3D BN/Ag HNMs for the methanol oxidation reaction, i.e., for the gas phase reaction. In the latter case, the usage of 3D BN NPs appears to be preferential because of higher specific surface area and gas permeability.

### IR spectroscopy

**Figure 6:**
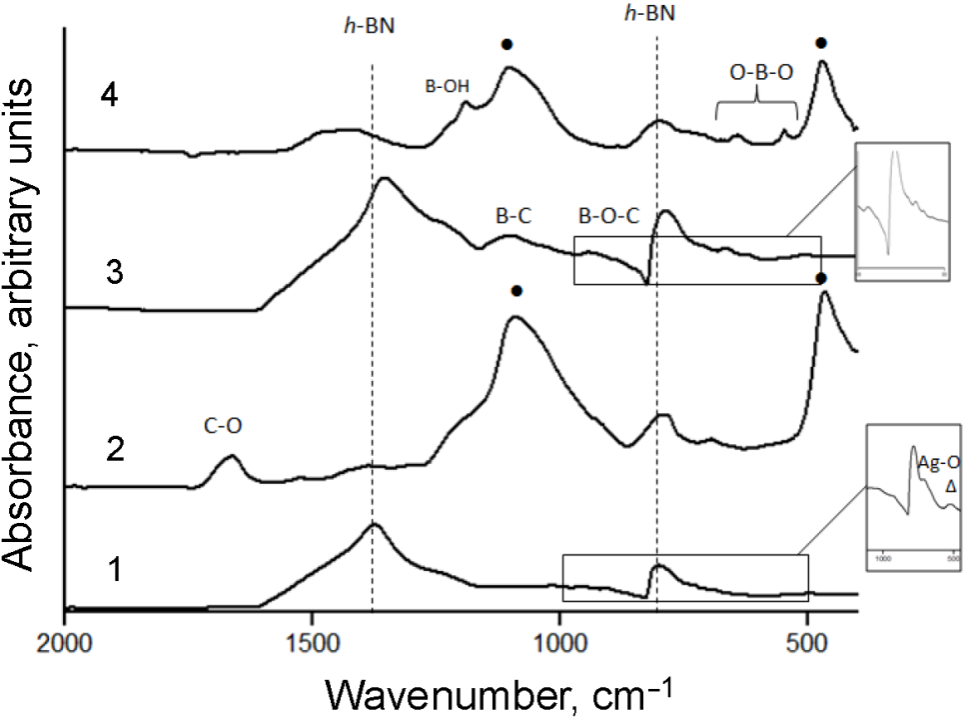
FTIR spectra of BN/Ag HNMs produced via CVD (1, 2) and AgNO_3_ decomposition (3, 4) before (1, 3) and after catalytic activity tests (2, 4). • Si–O. In both types of the BN/Ag HNMs Ag NPs are seen to be partly oxidized.

In order to trace the chemical state of the BN/Ag NH surfaces, the samples before and after catalytic activity tests were characterized by IR spectroscopy. The results are presented in Figure 6.

Both types of the pristine BN/Ag HNMs are characterized by the two main features in their FTIR spectra: a sharp low wavenumber peak at 769 cm^−1^ and a broad high wavenumber mode at 1359 cm^−1^ (spectra 1 and 3 in Figure 6), which correspond to out-of-plane B–N–B bending and in-plane B–N stretching vibrations, respectively [18–19]. The absorbance bands observed in the range of 800–1100 cm^−1^ were assigned to B–C and B–O–C bonds [20–22]. A small peak at 535 cm^−1^ in the FTIR spectrum of CVD BN/Ag HNMs (Figure 6 (inset)) indicates the oxidized state of Ag [23]. The partial oxidation of Ag during the CVD process appeared to occur during the cooling stage. Considering the XPS results described above, we can conclude that the Ag NPs were in an oxidized state in both types of BN/Ag HNMs.

After catalytic activity tests (Figure 6, spectra 2 and 4), two strong peaks were observed at 1104 cm^−1^ and 471 cm^−1^ in both CVD and UV BN/Ag samples. These were assigned to Si–O groups [24]. The formation of silicon oxide can be explained by a chemical reaction between the molten B_2_O_3_ phase in the BN/Ag HNMs and the quartz pellets. In addition, the UV BN/Ag sample revealed absorption bands within the 500–700 cm^−1^ range (Figure 6, spectrum 4), which can be ascribed to the deformation vibrations of the O–B–O groups [25–26]. Thus, the results indicate the appearance of the B_2_O_3_ phase after the catalytic activity tests of the UV BN/Ag HNMs, being in agreement with the XPS data. In contrast, the O–B–O bands were not observed in CVD BN/Ag HNMs, hereby indicating that BN-shielded Ag NPs were not oxidation catalysts for BN. This observation further supports our assumption that the oxidation processes expressed by reactions (2) and (3) occur due to the catalytic effect of uncovered Ag NPs. The FTIR spectrum of CVD BN/Ag HNMs after catalytic tests showed an asymmetric maximum at 1664 cm^−1^ due to the presence of C–O groups [27]. Their origin appears to be due to the oxidation of carbon impurities present in the CVD reactor within a thermal insulating coating.

### Structure of BN/Ag hybrid nanomaterials after catalytic activity tests

**Figure 7:**
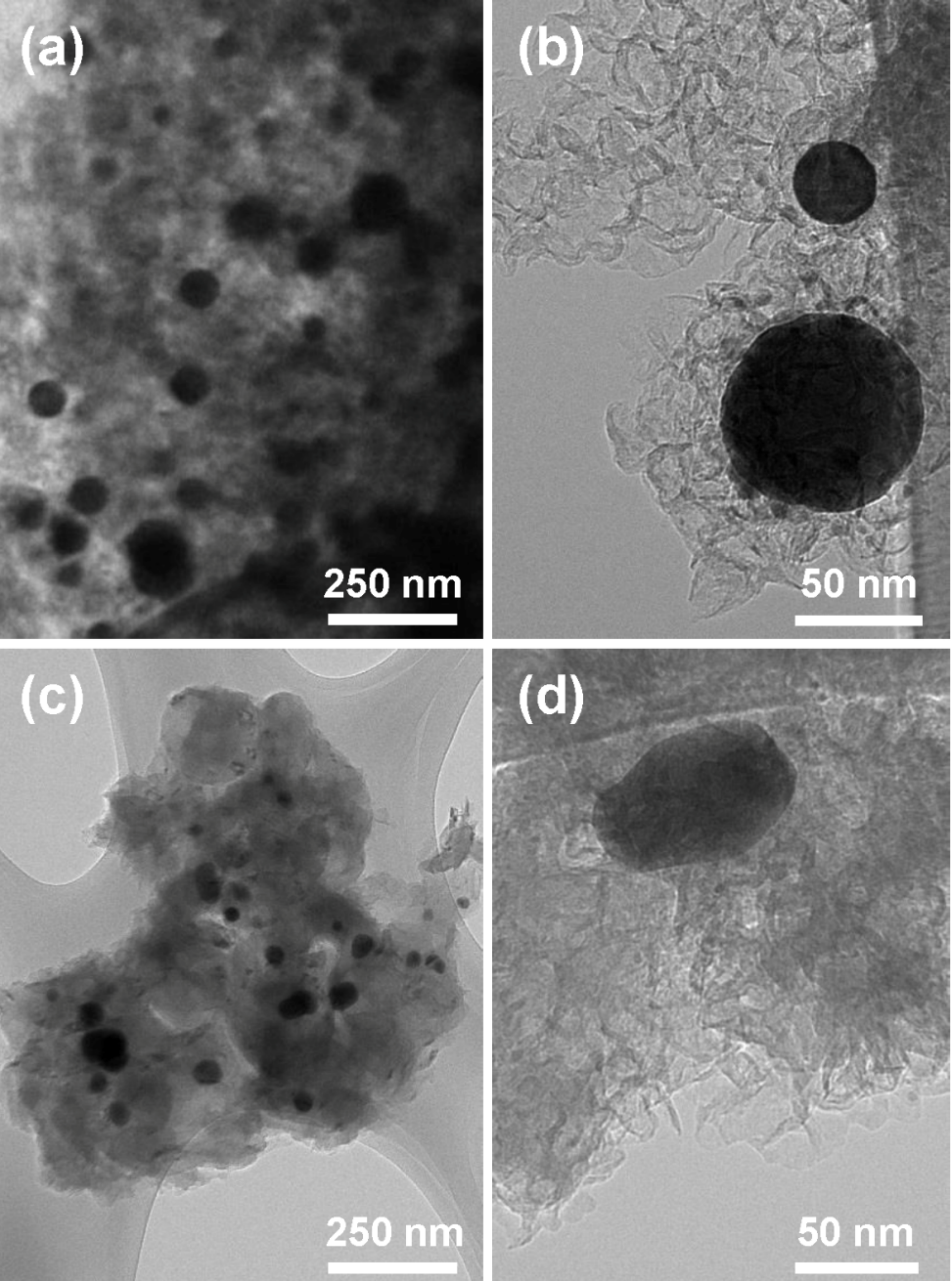
TEM images of BN/Ag hybrid nanomaterials produced via CVD (a, b) and UV decomposition of AgNO_3_ (c, d) after catalytic activity tests.

TEM images of BN/Ag HNMs after catalytic activity tests terminated at 600 °C are presented in Figure 7a–d.

It can be seen that the size of Ag NPs significantly increased from 5–15 nm to 15–120 nm. Since Ag and BN are two immiscible components of BN/Ag HNMs, the rise in temperature can lead to an increase of Ag atom mobility and coalescence of Ag NPs. The activation energy for surface diffusion of Ag was shown to depend on the type of a substrate (67 kJ/mol (Ni), 73 kJ/mol (Cu), and 100–155 kJ/mol (TiCaPCON film)), but was always lower than the activation energy for bulk self-diffusion in Ag (191 kJ/mol) [28]. A significant increase in the diffusion mobility of Ag atoms within a ceramic film was already observed at 230–240 °C [29]. Surprisingly, however, the obtained results are in contradiction with the thermal stability data reported in the previous section. At present, we can only assume that the methanol oxidation reaction or reaction products somehow affect the size of Ag NPs. Thus, additional studies are needed to uncover the exact Ag NP growth mechanism. Finally note that the “pompon”-like structure of BN NPs was fully preserved after catalytic activity tests.

### Ag^+^ ion release

**Figure 8:**
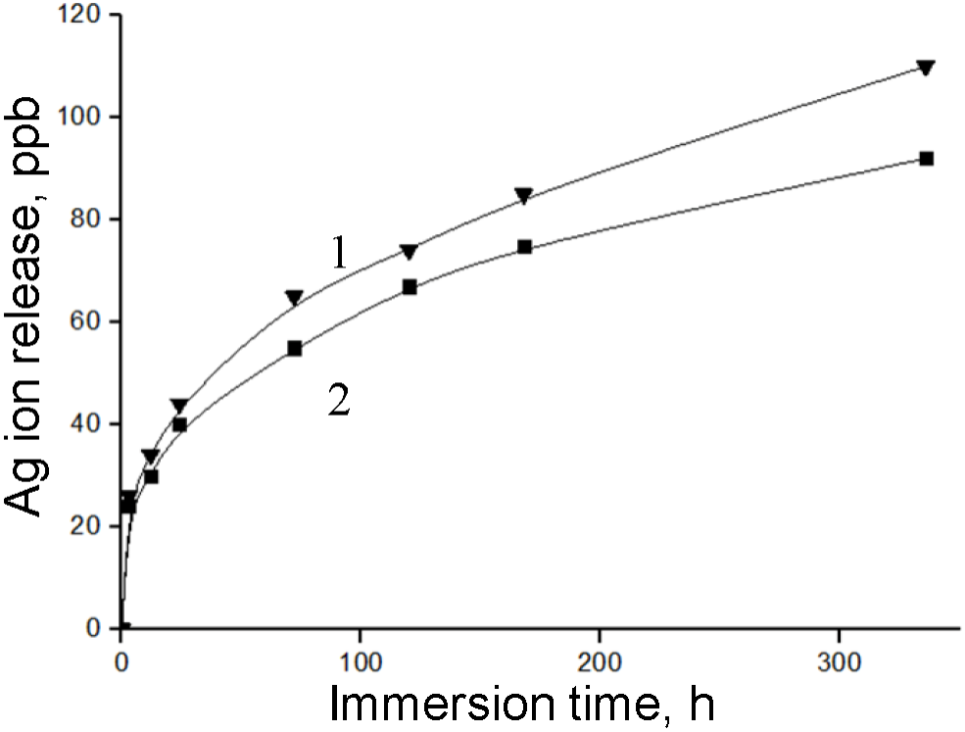
ICP–MS results of Ag^+^ ions leaching out of the BN/Ag hybrid nanomaterials produced via AgNO_3_ decomposition (1) and CVD (2).

Figure 8 compares the results of Ag^+^ ion release from BN/Ag HNMs obtained via CVD and UV decomposition of AgNO_3_.

Both samples demonstrated a sustained ion release over 14 days and similar ion release kinetics. During the first 3 h, approximately 25 ppb of Ag^+^ ions were released, after this, the Ag^+^ ion leaching gradually slowed down. The average rate of Ag^+^ ion release was 1.66–1.75 ppb/h (during the first day), 0.3–0.48 ppb/h (on days 2 and 3), and 0.15–0.25 ppb/h (on days 4 and 5). Note that, in the case of UV BN/Ag HNMs, the Ag^+^ ion concentration was 5–17% higher compared with the CVD BN/Ag HNMs, whose surface was covered with an *h-*BN layer. For the whole duration of testing (14 days), the maximum concentration of Ag^+^ ions was 90 ppb (CVD BN/Ag HNMs) and 110 ppb (UV BN/Ag HNMs).

### Antibacterial activity

**Figure 9:**
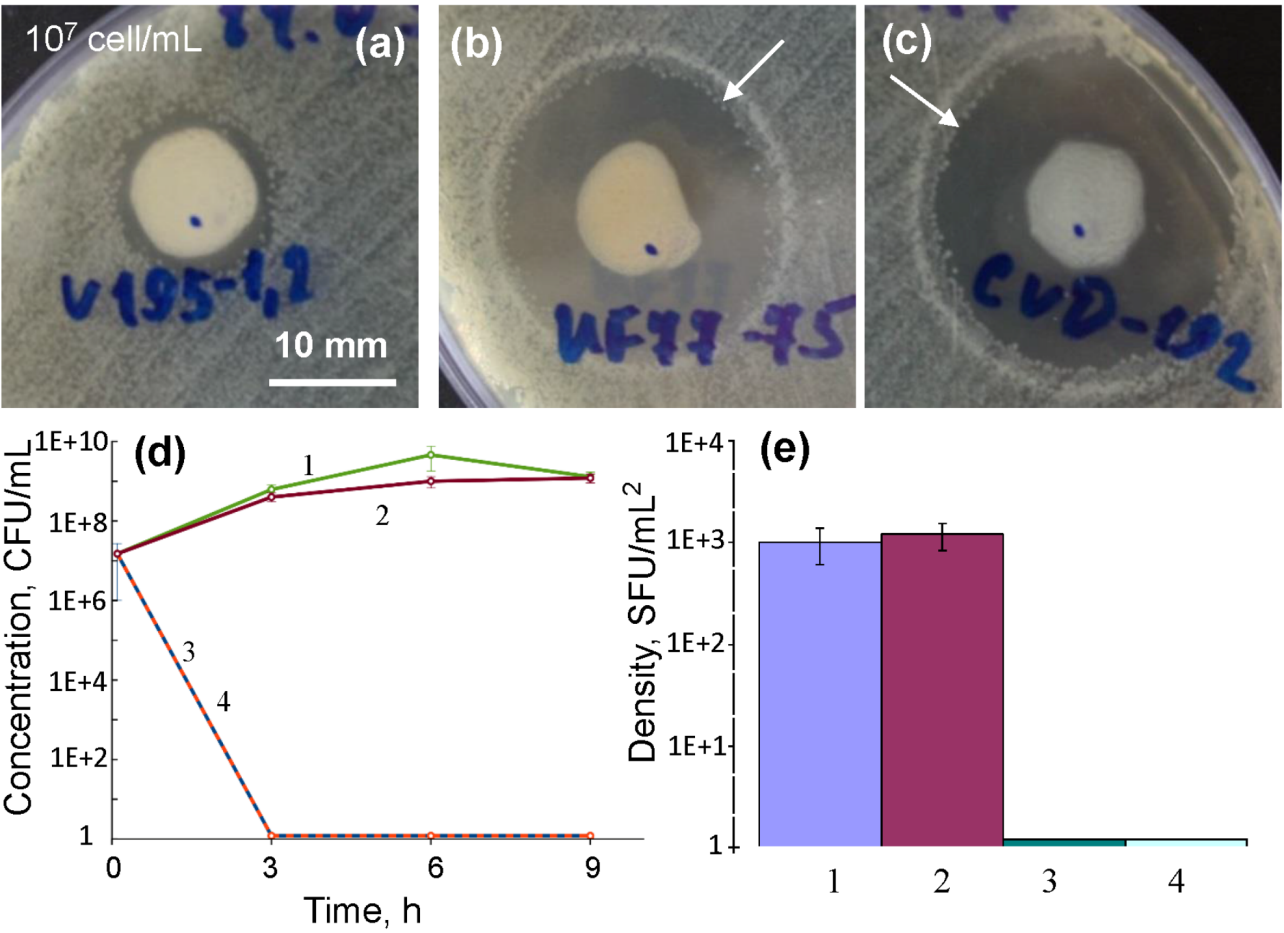
Antibacterial activity of BN/Ag NH samples against *E. coli* K-261. Zones of bacterium inhibition around tested samples (a) BN NPs, (b) UV BN/Ag HNMs, (c) CVD BN/Ag HNMs, changes of bacterium concentration vs time (d), and density of bacterial culture in a biofilm (e). 1 – control, 2 – BN NPs, 3 – UV BN/Ag HNMs, 4 – CVD BN/Ag HNMs. Zones with reduced cell concentration just behind the inhibition zones are shown by arrows.

The antibacterial activity of BN/Ag HNMs obtained via CVD was first studied using the inhibition zone method. The diameter of all discs with tested samples was 11 mm. The pristine BN sample did not show any noticeable effect on the inhibition of *E. coli* colony growth (Figure 9a). In contrast, Ag ions leaching out of the samples demonstrated a significant inhibition effect on the growth of *E. coli* K-261 bacteria around BN/Ag HNMs (Figure 9b and 9c). The width of inhibition zone increased from 1.5 mm (BN) to 6 mm (UV BN/Ag HNMs) and 7–8 mm (CVD BN/Ag HNMs). Note that in both Ag-containing samples additional zones, 1–1.5 mm wide, with a reduced cell concentration were observed just behind the inhibition zones (shown by arrows). This effect can be associated with the appearance of bacterial cell resistivity to low Ag^+^ ion concentration.

Then, the antibacterial activity of the BN/Ag HNMs was evaluated against planktonic *E. coli* bacteria. After incubation for 3 h, the number of CFUs in the presence of both types of BN/Ag HNMs decreased to zero (Figure 9d, lines 3 and 4), whereas control and BN NP samples did not show any antibacterial effect (Figure 9d, curves 1 and 2).

In order to estimate the antibacterial activity of the BN/Ag HNMs at the early stage of biofilm formation, the coupon method was used. The obtained results show that, unlike the control sample, there is no biofilm formation on the surface of CVD and UV BN/Ag HNMs (Figure 9e). Thus, our data demonstrate that both types of BN/Ag HNMs possess a strong antibacterial effect against *E*. *coli* bacteria and inhibit the early stage of biofilm formation.

Despite a broad spectrum of antibacterial activity, the wide application of Ag NPs is restricted by their poor dispersion stability and high tendency to agglomeration. This reduces their specific surface areas, dissolution rate and, eventually, antibacterial efficiency. Therefore, it is extremely important to use supports which could not only improve the colloidal stability of Ag NPs but also improve their antibacterial characteristics. A wide range of materials, such as graphene oxide [30–31], carbon nanotubes [32], SiO_2_ [33], Fe_3_O_4_ [34], ZnO [35], CuO [36], TiO_2_ [37] and others, have been tested as the supports for Ag NPs. Compared with the previous studies, our approach possesses the notable advantages owing to the particle spherical shape, their optimal size and petal-like surfaces resulting in larger contact areas. Nanoparticles larger than 200 nm are sequestered in liver, spleen, and kidneys, while those smaller than 100 nm may leave the blood vessels through fenestrations in the endothelial lining [38]. Recent studies have revealed that BN NPs are not toxic and can be additionally saturated with chemotherapeutic agents for multifunctional biological applications [39]. Therefore, our results open up new possibilities for the production of cost-effective, scalable, and biologically safe BN/Ag HNMs with pronounced antibacterial and catalytic characteristics for future medical and advanced “green” energy applications.

Summing up, all the above described tests and structural studies have confirmed that synthesised BN/Ag HNMs have high catalytic and antibacterial activity. It should be mentioned that in some previous works it has been already reported that hybrid nanomaterials based on Ag NPs on different supports can be effective catalysts and antibacterial agents. Most of these hybrid nanomaterials have similar average sizes and specific surface areas of Ag NPs [5,10,12,14,40], but the thermal stability of Ag based hybrid nanomaterials has not been studied before.

Nevertheless, the prime novelty of the present work is in the designing of advanced supports – petal-like BN nanoparticles. Utilization of these nanostructures opens up new opportunities to improve the performance of hybrid nanomaterials. Many techniques for surface modification [4] and band-gap engineering [41–42] were described in the past few years. This allows for a control of size and homogeneity of Ag NPs, adhesion and charge distribution on the BN/Ag interface. All these properties have a significant influence on the catalytic and antibacterial performance of hybrid nanomaterials. For example, our preliminary experiments have shown that partial oxidation of BN NPs allows us to reduce the size of Ag NPs and to decrease the temperature of 100% methanol conversion down to 150 °C what is significantly lower compared to all known hybrid nanomaterials.

## Conclusion

Hybrid BN/Ag NPs were fabricated using two methods: (i) chemical vapour deposition (CVD) of BN NPs in the presence of Ag vapours, and (ii) ultraviolet decomposition of AgNO_3_ in a suspension of CVD-obtained BN NPs. Structural studies revealed that the hybrid nanomaterials had consisted of numerous BN nanosheets self-organized in solid NPs with an average size of 50–200 nm and evenly covered with partially oxidized Ag NPs having diameters of 5–15 nm. The nanomaterials obtained via UV decomposition of AgNO_3_ demonstrated much better catalytic activity compared with the hybrid nanomaterials fabricated via CVD because the major part of Ag NPs in the latter case was covered with the layers of *h*-BN, thereby reducing their catalytic activity. On the other hand, CVD BN/Ag HNMs with encapsulated Ag NPs demonstrated a possibility to create an “eternal catalyst”. The best catalytic characteristics (100% methanol conversion at 350 °C) were achieved for the UV BN/Ag HNMs without preliminary activation stage. Our results also confirmed that both types of BN/Ag HNMs possess a strong antibacterial effect against *E. coli* K-261.

## Experimental

### Synthesis of BN NPs and BN/Ag hybrid nanomaterials

Raw BN NPs were produced using a boron oxide CVD process from a mixture of FeO, MgO, and B powders (a weight ratio 150:28:75) via a reaction of boron oxide vapour and flowing ammonia in the vertical induction furnace, as described elsewhere [43].

For the synthesis of BN/Ag HNMs, a piece of pure Ag foil was placed in the hot zone of the CVD reactor during BN NPs preparation. As a result, Ag vapors were simultaneously condensed along with the nucleation and growth of the BN NPs. This technique allows us to produce grams of BN/Ag HNMs in a single experimental run (referred to as CVD BN/Ag HNMs). To prepare BN/Ag HNMs via UV decomposition of AgNO_3_ (abbreviated as UV BN/Ag HNMs), we have dispersed 100 mg of raw BN NPs in 50 mL of polyethylene glycol 400 (PEG-400) using an ultrasonic homogenizer Bandelin HD2200, and then 75 mg of AgNO_3_ were added to the BN NP/PEG suspension. Next, the mixture was exposed to an UV lamp (185 nm) for 30 min during magnetic stirring, and then washed with distilled water and dried.

### Structural characterization

The microstructure and morphology of the BN/Ag HNMs were studied by means of high-resolution transmission electron microscopy (HRTEM), high-angular dark field scanning TEM (HADF-STEM) imaging and energy-dispersive X-ray spectroscopy (EDX) mapping using a JEM-2100 microscope (JEOL) equipped with an energy-dispersive X-ray spectrometer (Oxford Instruments) and a STEM detector (JEOL). FTIR spectroscopic measurements of BN/Ag HNMs were carried out in the attenuated total reflectance (ATR) mode using a Vertex 70v vacuum spectrometer (Bruker) in the range of 400–4000 cm^−1^ with a resolution of 4 cm^−1^.

The chemical composition of the sample surfaces was characterized by X-ray photoelectron spectroscopy (XPS) using an Axis Supra (Kratos Analytical) spectrometer. The powder samples were attached to the carbon tape covered with glue. The maximum lateral dimension of the analyzed area was 0.7 mm. To avoid differential charging of samples, the spectra were acquired with charge neutralization. The spectra were subsequently normalized by shifting the hydrocarbon component to 285.0 eV and acquired at the pass energy of 40 eV. Due to the carbon signal coming from the tape, all spectra exhibited a strong carbon signal. The oxygen peak was also enhanced due to the glue on the carbon tape surface. In order to compensate the oxygen coming from the tape, the C 1s spectrum was fitted and the calculated amounts of **C**(O)O and **C**–O arrangements were used to calculate the amount of oxygen related to the carbon tape. Fitting of XPS C 1s, N 1s, and O 1s was carried out using the CasaXPS software (version 2.3.17) after subtraction of the Shirley-type background employing Gaussian–Lorentzian (G–L) peaks with the fixed G–L percentage of 30%. The values of binding energies (BEs) of C 1s, N 1s, O 1s, B 1s, and Ag 3d components were taken from the literature [44–47].

### Ag^+^ ion release

The rates of Ag^+^ ion release from BN/Ag HNMs were studied by means of inductively coupled plasma mass spectrometry (ICP–MS) using an X-Series II unit. 15 mg powder mixtures of BN/Ag HNMs were immersed in flasks filled with 40 mL of distilled water at room temperature and then sonicated for 1 min. To analyze the Ag^+^ ion concentration, 1 mL of the solution was collected after 3 h, 12 h, and 1, 3, 5, 7, and 14 days.

### Thermal stability of BN/Ag hybrid nanomaterials

The thermal stability of BN/Ag HNMs was first evaluated under in situ heating inside the TEM column using a Gatan heating holder. The structures of as-fabricated samples were compared with those annealed at 700 °C. In order to model the temperature and environmental conditions during catalytic activity tests, the BN/Ag HNMs samples were preliminary annealed in air at 600 °C for 1 h and then analyzed inside TEM during additional heating.

### Catalytic performance

Catalytic activity tests were carried out for both types of the BN/Ag HNMs. The samples were tested with respect to a methanol oxidation reaction in a fixed-bed continuous-flow reactor at the atmospheric pressure. 40 mg (0.1 cm^3^) of BN/Ag HNMs were mixed with 0.4 cm^3^ of quartz pellets (≈400 μm in size) and placed in an U-tube. Before the activity measurements, the catalysts were activated in a flow of 10% O_2_/N_2_ gas mixture at the total flow rate of 67 mL/min. After the activation at 600 °C, the furnace was cooled down to room temperature and methanol was added to the gas flow using a saturator. The concentration of methanol was 3.5 vol % and the total gas flow rate was kept constant at 67 mL/min, i.e., the gas feed rate was 40000 h^−1^. The catalytic activity was measured in the temperature range from 50 °C up to the temperature of the full methanol oxidation. The gas composition was analyzed by a ThermoStar mass-spectrometer (Pfeiffer Vacuum). The conversion of methanol was calculated based on the intensity of the peak at 31 amu. For comparison, catalytic activity tests of the BN/Ag HNMs obtained via UV decomposition of AgNO_3_ without preliminary activation stage were carried out.

### Antibacterial activity

The antibacterial activity of BN/Ag HNMs was studied against *Escherichia coli* K-261 bacteria. The bacterial culture was cultivated on solid nutrient Müller-Hinton Agar (MHA) or in nutrient broth. For primary screening, we used the cap-plate method (agar diffusion test). Bacterial suspension (10^7^ cells/mL) was prepared in a physiological solution. Then, 0.1 mL of the tested bacterial culture was added onto the MHA growth medium. Raw BN NPs (0.07 g/L) were ultrasonically treated in a 3% ethanol solution using a Soniprep 150 homogenizer (MSE Ltd.) for 5 min. Then 20 µL of the BN NP suspension was placed on the agar surface and incubated at 37 °C for 24 h. To evaluate the antibacterial activity of BN/Ag HNMs against planktonic *E. coli*, 1 mL of bacterial culture was placed into 25 mL of nutrient broth and stirred at 150 rpm for 1 min. Then, 5 mL of the suspension was inoculated in 25 mL of nutrient broth, mixed with 500 µL of BN NP suspension and cultivated at 37 °C for 8 h. After 3, 6, and 9 h of incubation, 60 µL of bacterial suspension was dispersed onto a solid nutrient agar in sterile Petri dishes and the number of surviving colony forming units (CFUs) in each Petri dish was counted. In order to estimate the antibacterial activity of the BN/Ag HNMs at the early stage of biofilm formation, the coupon method was used. Three standard plastic coupons, 300 mm^2^, were immersed into *E. coli* bacterial culture together with the BN/Ag HNMs at 37 °C for 8 h. After the tests, the coupons were removed, rinsed with 10 mL of physiological solution, and finally sonicated for 2 min to remove bacterial cells from the surface of coupons. Then 60 mL of resulting suspension was selected and a number of CFUs was counted.

## Supporting Information

File 1 Evaluation of the specific surface area of Ag NPs, histograms of Ag NPs size distribution and TEM microphotograph of CVD BN/Ag hybrid nanomaterials.
