# Supplementary material for: BN/Ag hybrid nanomaterials with petal-like surfaces as catalysts and antibacterial agents
Source: Beilstein J Nanotechnol. 2018 Jan 23;9:250–61. doi: 10.3762/bjnano.9.27 (PMC5789442; doi:10.3762/bjnano.9.27)
Supplement: File 1 — Evaluation of the specific surface area of Ag NPs, histograms of Ag NPs size distribution and TEM microphotograph of CVD BN/Ag hybrid nanomaterials. [file Beilstein_J_Nanotechnol-09-250-s001.pdf]

Supporting Information

for

BN/Ag hybrid nanomaterials with petal-like surfaces as catalysts and antibacterial agents

Konstantin L. Firestein[1,2]*, Denis V. Leybo[1], Alexander E. Steinman[1], Andrey M. Kovalskii[1], Andrei T. Matveev[1], Anton M. Manakhov[1], Irina V. Sukhorukova[1], Pavel V. Slukin[3], Nadezda K. Fursova[3], Sergey G. Ignatov[3,4], Dmitri V. Golberg[2,5] and Dmitry V. Shtansky[1]*

Address: [1]National University of Science and Technology "MISIS", Leninsky prospect 4, Moscow, 119049, Russian Federation, [2]School of Chemistry, Physics and Mechanical Engineering, Science and Engineering Faculty, Queensland University of Technology (QUT), 2nd George st., Brisbane, QLD 4000, Australia, [3]State Research Center for Applied Microbiology and Biotechnology, Obolensk, Moscow Region 142279, Russian Federation, [4]Moscow State University, Department of Geocryology, Moscow 119992, Russian Federation and [5]World Premier International Center for Materials Nanoarchitectonics (WPI-MANA), National Institute for Materials Science (NIMS), Tsukuba, Namiki 1, Ibaraki 3050044, Japan

Email: Konstantin L. Firestein - konstantin.faershteyn@qut.edu.au; Dmitry V. Shtansky - shtansky@shs.misis.ru;

* Corresponding author

Evaluation of the specific surface area of Ag NPs, histograms of Ag NPs size distribution and TEM microphotograph of CVD BN/Ag hybrid nanomaterials

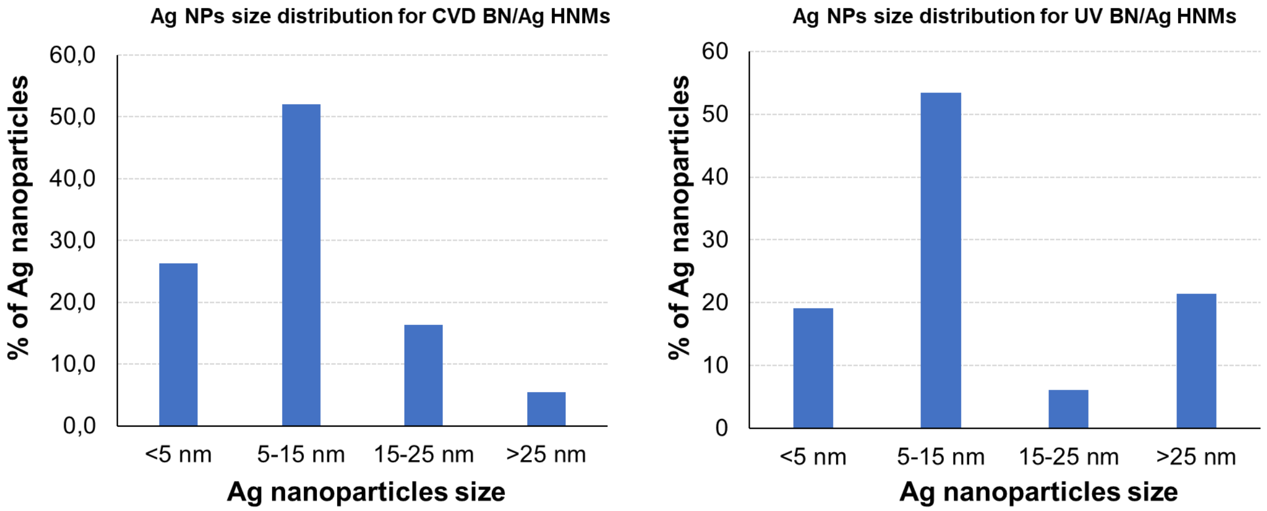

Figure S1: Histograms of the Ag NP size distribution for CVD BN/Ag hybrid nanomaterials (a) and UV BN/Ag hybrid nanomaterials (b).

Evaluation of the specific surface area of Ag NPs.

Considering that all silver NPs have a shape close to spherical the specific surface area of the Ag NPs could be calculated using the following equation:

$$SSA = \frac{S}{m} = \frac{S}{\rho \cdot V} = \frac{4\pi R^2}{\rho \cdot 4/3\pi R^3} = \frac{3}{\rho \cdot R} \qquad (1)$$

Where ρ is density of Ag; and *R* is an average radius of Ag NPs.

The average radius can be estimated from TEM images using secant method. For this 5–7 random straight lines are drawn through the TEM micrograph, then sizes of NPs lying on each line are measured (Figure S2). This was repeated for 5 micrographs for each sample. The average radius was calculated as following:

$$R_{av} = \frac{\sum_n R}{n} \qquad (1)$$

The calculated average radius of Ag NPs was 7 nm for CVD BN/Ag hybrid nanomaterials and 11 nm for UV BN/Ag hybrid nanomaterials. Specific surface areas of the Ag NPs were respectively 4.3×10^5 cm^2/g and 2.7×10^5 cm^2/g.

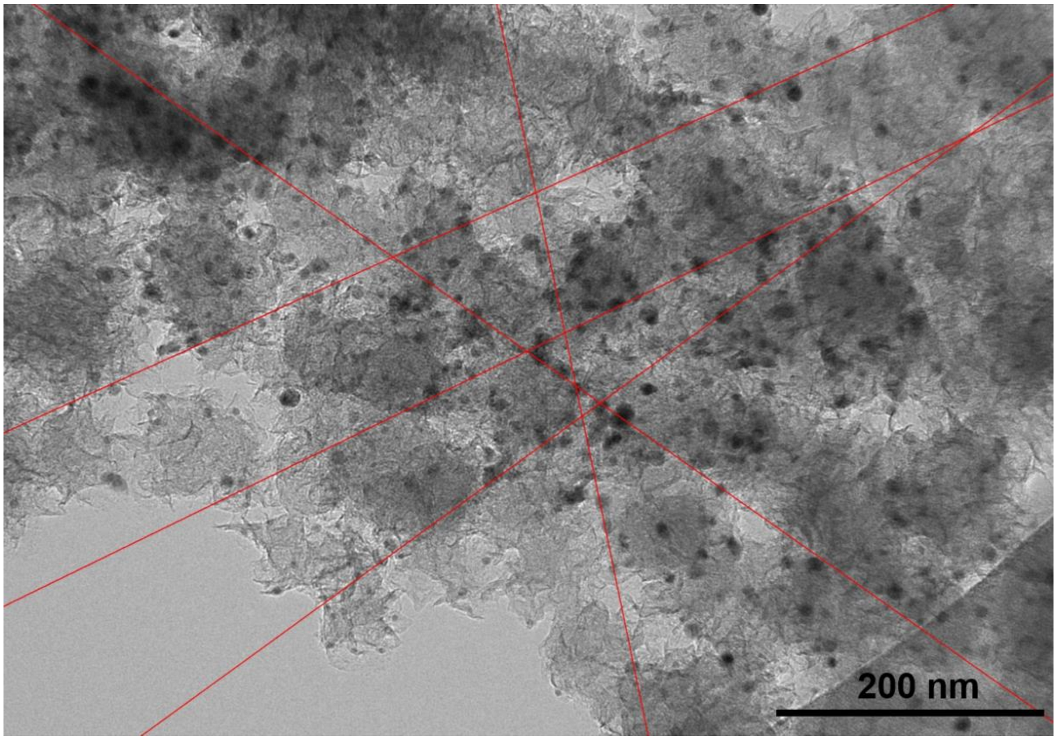

Figure S2: TEM microphotograph of CVD BN/Ag hybrid nanomaterials with randomly drawn straight lines.
